# The Oncolytic Virotherapy Era in Cancer Management: Prospects of Applying H-1 Parvovirus to Treat Blood and Solid Cancers

**DOI:** 10.3389/fonc.2017.00093

**Published:** 2017-05-12

**Authors:** Assia L. Angelova, Mathias Witzens-Harig, Angel S. Galabov, Jean Rommelaere

**Affiliations:** ^1^Department of Tumor Virology, German Cancer Research Center, Heidelberg, Germany; ^2^Department of Hematology, Oncology and Rheumatology, University Hospital Heidelberg, Heidelberg, Germany; ^3^Department of Virology, The Stephan Angeloff Institute of Microbiology, Bulgarian Academy of Sciences, Sofia, Bulgaria

**Keywords:** oncolytic virotherapy, oncolytic H-1 parvovirus, glioblastoma, pancreatic ductal adenocarcinoma, oncolytic (parvo)virotherapy of hematological malignancies, diffuse large B-cell lymphoma, cutaneous T-cell lymphoma

## Abstract

Non-Hodgkin lymphoma (NHL) and leukemia are among the most common cancers worldwide. While the treatment of NHL/leukemia of B-cell origin has much progressed with the introduction of targeted therapies, few treatment standards have been established for T-NHL/leukemia. As presentation in both B- and T-NHL/leukemia patients is often aggressive and as prognosis for relapsed disease is especially dismal, this cancer entity poses major challenges and requires innovative therapeutic approaches. In clinical trials, oncolytic viruses (OVs) have been used against refractory multiple myeloma (MM). In preclinical settings, a number of OVs have demonstrated a remarkable ability to suppress various types of hematological cancers. Most studies dealing with this approach have used MM or B- or myeloid-cell-derived malignancies as models. Only a few describe susceptibility of T-cell lymphoma/leukemia to OV infection and killing. The rat H-1 parvovirus (H-1PV) is an OV with considerable promise as a novel therapeutic agent against both solid tumors (pancreatic cancer and glioblastoma) and hematological malignancies. The present perspective article builds on previous reports of H-1PV-driven regression of Burkitt’s lymphoma xenografts and on unpublished observations demonstrating effective killing by H-1PV of cells from CHOP-resistant diffuse large B-cell lymphoma, cutaneous T-cell lymphoma, and T-cell acute lymphoblastic leukemia. On the basis of these studies, H-1PV is proposed for use as an adjuvant to (chemo)therapeutic regimens. Furthermore, in the light of a recently completed first parvovirus clinical trial in glioblastoma patients, the advantages of H-1PV for systemic application are discussed.

## Introduction

### Viruses and Human Health, a Two-Edged Sword: Chronology of Virus Rehabilitation

*1898: viruses are discovered as “minute living things capable of reproducing themselves*.” After the pioneering work of Adolf Eduard Mayer, Dmitri Ivanovsky, and Martinus Beijerinck, two German researchers, Friedrich Loeffler and Paul Frosch, were the first to contradict the “contagium vivum fluidum” (contagious living fluid) hypothesis to define a virus (the foot-and-mouth disease virus) as a tiny particle and to suggest that “the causative agents of numerous other infectious diseases of man and animals may also belong to this group of minute organisms” (1). Thus, at the beginning of the 20th century, the door opened to a new and exciting research area: virology.

*Twentieth century: viruses as triggers of human infectious diseases*. In the course of the 20th century and as predicted by Loeffler and Frosch, viruses were identified as the unquestionable causative agents of many human infectious diseases, from yellow fever (2), rabies (3), and poliomyelitis (4) to the acquired immunodeficiency syndrome (5). And this was not the end of the story: new disease-causing viruses, such as human bocaviruses, continue to emerge (6). It never rains but it pours…

*Further bad news: viruses and human cancer*. In addition to their vicious role as causative agents of numerous human infectious diseases, viruses are also involved in cancer development. This was first demonstrated at the beginning of the 20th century. Some 15–20% of all human cancers are attributed to viruses, notably Epstein–Barr virus, papilloma viruses, hepatitis B and C viruses, human herpesviruses, and human T-lymphotropic virus 1 (7). The molecular mechanisms underlying virus-induced carcinogenesis are diverse and complex. In addition to causing direct effects such as induction of genomic instability, DNA damage, and viral oncogene-triggered cell transformation (8, 9), oncogenic viruses can establish a chronic infection allowing them to escape from the host’s immune system while producing proteins that control cell death and proliferation. Chronic infection also leads to inflammatory reactions promoting cancer development (10). In nasopharyngeal cancer, certain lymphomas, cervical cancer, liver cancer, Kaposi’s sarcoma, and human adult T-cell leukemia/lymphoma, malignant transformation is likely to be initiated by host cell infection by an oncogenic virus. And yet…

*Two sides to every coin: viruses have a bad side and an “oncolytic” side*. Breakthrough observations at the start of the 20th century and findings peaking in the 1950s made it clear that “severe (virus) infections may on occasion favorably modify the course of far-advanced neoplastic disease…” (11). A significant drop in leukocyte counts associated with some clinical improvement was documented in children diagnosed with acute lymphoblast leukemia (ALL) having simultaneously acquired a varicella virus infection (11). At least five cases showing Hodgkin’s disease regression after measles virus infection were described (12–14). Similar observations were made in patients having developed viral hepatitis during Hodgkin’s lymphoma progression (15). In 1971, Bluming and Ziegler published a case report on Burkitt’s lymphoma (BL) regression associated with measles virus infection (16). Today, more than a century after the first report on virus infection-associated clinical remission in cancer patients, virotherapy with oncolytic viruses (OVs) is the focus of a rapidly growing research field. Studies in this field have brought convincing evidence that oncolytic virotherapy, alone or in combination with surgery, chemotherapy, or radiotherapy, may significantly impact cancer mortality and improve patients’ quality of life.

### Oncolytic Viruses As Anticancer Tools: From Bench to Clinical Trials

Oncolytic viruses form a diverse biological group whose members belong to at least 10 different virus families, contain either an RNA or a DNA genome, and vary considerably as regards genome size, particle complexity, and natural host preferences (17). OVs naturally possess? or are engineered to acquire the capacity to selectively infect, replicate in, and destroy tumor cells (oncolysis) while sparing their normal counterparts (17, 18). Multiple factors explain this oncoselectivity: altered expression by tumor cells of virus entry receptors and/or intracellular permissiveness factors, rapid tumor cell division and high metabolic activity, deficient antiviral type I interferon responses in tumor cells, etc. (19). Furthermore, there is mounting evidence that OV infection of tumor cells induces an immunogenic process, with neo-antigen recognition and establishment of specific antitumor immune responses (20). The remarkable potential of OVs as cancer therapeutics has been well documented in a number of preclinical studies, and the resulting knowledge has been translated into an expanding wave of clinical trials (21, 22). In 2015, talimogene laherparepvec was the first OV to receive FDA approval as an anticancer drug (23) based on the fact that this herpes simplex virus type 1-based oncolytic immunotherapy has demonstrated therapeutic benefit against metastatic melanoma in a phase III clinical trial (24). In 2016, there were about 40 OV-based clinical trials recruiting cancer patients (19).

## H-1 Parvovirus (H-1PV) Against Pancreatic Cancer and Glioma: The Brave Little Cancer Fighter

With a particle diameter of only 22 nm, the non-enveloped ssDNA-containing H-1 parvovirus is the smallest of the OVs. Its natural host is the rat. Humans are not naturally infected with H-1PV, no firm association between this virus and any human disease has been established, and no preexisting H-1PV immunity has been detected in the human population (25). Failure to observe any virus-related pathogenic effects in two early studies of H-1PV administration to human cancer patients (26, 27) prompted further therapy-oriented H-1PV research. Considerable preclinical evidence has accumulated over the last 30 years [reviewed in Ref. (28–30)] providing straightforward proof that H-1PV has broad oncosuppressive potential. In particular, pancreatic carcinoma and glioblastoma have attracted major attention as parvovirotherapy targets. In the respective preclinical models, efforts have been made to unravel the mechanisms and improve the efficacy of H-1PV treatment.

### Pancreatic Cancer

Pancreatic ductal adenocarcinoma (PDAC) is a highly aggressive tumor, often unresectable at the time of initial diagnosis. Median overall survival is only 6–9 months. As current therapies for PDAC patients fail to improve significantly their quality of life and to prolong survival (31), it is urgent to develop novel curative strategies. Extensive work by our team on H-1PV-based virotherapy for PDAC has yielded the following key findings: (i) H-1PV efficiently kills PDAC cells, including gemcitabine-resistant ones (32); (ii) H-1PV infection of pancreatic cancer cells results in active cathepsin B translocation to the cytosol (32) and in extracellular HMGB1 danger signaling (33); (iii) some predictive markers of PDAC permissiveness to H-1PV infection and lysis, e.g., SMAD4, have been identified (34); (iv) in an orthotopic PDAC model, H-1PV causes tumor regression and prolongs animal survival, without affecting bone marrow activity, liver function, or kidney function (32); (v) H-1PV-induced tumor suppression is potentiated under conditions of gemcitabine pretreatment (the current gold standard in pancreatic cancer therapy) (32); (vi) H-1PV oncosuppressive effects involve the participation of immune cells, which become activated either after an abortive infection with the virus (35) or through induction of immunogenic factors such as NK cytotoxicity receptor ligands (36) in H-1PV-infected PDAC cells; (vii) the vaccination potential of H-1PV, in combination with IFN-γ, extends to the treatment of peritoneal carcinomatosis, an untreatable condition traditionally managed with palliative measures only (37). Current preclinical achievements and prospects for pancreatic cancer parvovirotherapy are summarized in Ref (38, 39).

### Glioma

Glioblastoma is the most aggressive human primary brain tumor. Life expectancy remains very poor, despite standard and alternative therapeutic attempts (40). Our team has shown that oncolytic H-1PV infection of human glioma cells results in efficient cell killing (41). High-grade glioma stem cell models are also permissive to lytic H-1PV infection (42). The cellular mechanism of virus-induced glioma cell killing has been elucidated and is based on active lysosomal cathepsin B translocation and accumulation in the cytosol of H-1PV-infected glioma cells but not normal cells (astrocytes) (43). Enhanced glioma cell killing has been observed when the virus was applied shortly after tumor cell irradiation, suggesting that this protocol might be translated to cases of non-resectable recurrent glioblastoma (44). In animal models, local, systemic, or intranasal administration of H-1PV has been found to cause regression of advanced tumors, virus replication being restricted to tumor tissues (45, 46). The favorable safety profile of local or systemic treatment with medical-grade GMP-produced H-1PV has been confirmed in a study using a permissive animal model (47, 48).

On the basis of the above preclinical evidence, the first phase I/IIa clinical trial (ParvOryx01) of an oncolytic parvovirus (H-1PV) in recurrent glioblastoma patients was launched in 2011 (49) and successfully completed in 2015. This trial, in addition to confirming the excellent safety and tolerability of H-1PV, yielded valuable observations, which strongly encourage further clinical development of this virus as an anticancer therapeutic. Particularly essential is the evidence suggesting that H-1PV (i) crosses the blood–brain barrier after systemic administration and (ii) may induce immunogenic conversion of the tumor microenvironment. In 2015, a second phase I/IIa trial was launched in inoperable metastatic PDAC patients. The outcome of this study is eagerly awaited.

Glioblastoma and pancreatic cancer are far from being the only tumor types sensitive to H-1PV-induced oncotoxicity, since it has also been demonstrated in preclinical models of breast, gastric, cervical (29), and colorectal (50, 51) cancer. H-1PV thus has the potential to treat not only brain and pancreatic but also a variety of other tumors.

## Oncolytic (Parvo)Viruses Against Hematological Malignancies

### Preclinical Experience

Lymphoma and leukemia are the two cancer types tightly associated with the dawn of the oncolytic virotherapy era. Later, however, they were superseded as oncolytic virotherapy targets by solid tumors, such as breast, ovarian, bladder, skin, colon, and lung carcinomas. Nevertheless, a substantial set of preclinical data shows that several OVs can selectively lyse hematopoietic stem cells or downstream blood cell lineages (Table 1). As shown in the table, the predominant preclinical model is multiple myeloma (MM), followed by leukemia/lymphoma of B-lymphoid, myeloid, or T-lymphoid origin. Myxoma virus, a poxvirus whose natural tropism is restricted to European rabbits and is non-pathogenic for other vertebrates, has been demonstrated to selectively induce apoptotic death in MM cells (52–54). MM has also been successfully targeted by a double-deleted vaccinia virus (55), adenovirus serotype 5 (56), coxsackievirus A21 (57), reovirus (58–60), vesicular stomatitis (VSV) virus (61), and measles virus (62–65). Furthermore, myxoma and VSV infections are oncotoxic to acute myeloid leukemia cells (66), while adeno- (67, 68), herpes- (69), reo- (70), VSV (71), and measles virus (72–76) are reported to induce killing/suppression of B- and T-lymphoma or leukemia-derived cells/xenografts.

**Table 1:** **Oncolytic viruses (OVs) targeting hematological malignancies: preclinical evidence**.

OV	Malignancy	Malignant cell type	Reference
**DNA viruses**			
Myxoma virus (*Poxviridae*)	MM, AML	Plasma, myeloid	(52–54)
Vaccinia virus (*Poxviridae*)	MM	Plasma	(55)
Adenovirus (*Adenoviridae*)	MM, lymphoma	Plasma, B-L	(56, 67, 68)
Herpes virus (*Herpesviridae*)	Lymphoma	B-L, T-L	(69)
**RNA viruses**			
CVA21 (*Picornaviridae*)	MM	Plasma	(57)
Reovirus (*Reoviridae*)	MM, lymphoma	Plasma, B-L	(58–60, 70)
VSV (*Rhabdoviridae*)	MM, AML, CLL	Plasma, myeloid	(61, 66, 71)
Measles virus (*Paramyxoviridae*)	MM, lymphoma, leukemia	Plasma, B-L, T-L	(62–65, 72–76, 87)
H-1PV (*Parvoviridae*)	Lymphoma, leukemia	B-L, T-L, myeloid	(77–79)

*MM, multiple myeloma; AML, acute myeloid leukemia; B-L, B-lymphocyte; T-L, T-lymphocyte; CVA21, coxsackie virus A21; VSV, vesicular stomatitis virus; CLL, chronic lymphocytic leukemia*.

First proofs of the capacity of H-1PV to infect and destroy human blood cancer-derived cells date back to the 1980s, when Faisst et al. screened for H-1PV permissiveness and killing a panel of BL, adult T cell leukemia-derived, and *in vitro*-transformed lymphoblastoid cell lines (77, 78). Further proof-of-concept was provided by Angelova et al., who showed that African and European BL cells, including those lacking CD20 and hence resistant to the CD20-targeting therapeutic rituximab, are highly susceptible to H-1PV-induced killing, in contrast to normal B lymphocytes from healthy donors. In a SCID mouse lymphoma model, a single intratumoral H-1PV injection was sufficient to cause full tumor suppression and disease-free survival for the whole period of observation (70 days). This striking oncosuppression was observed even when the virus was applied late after tumor initiation, so as to mimic an advanced disease stage (79).

### Clinical State of the Art

The rapid development of gene therapy and immune modulation approaches in recent years has led to greatly improving the management of many hematological cancer types. Several clinical trials are currently examining the effects of RNA interference, suicide gene therapy, and immune modulation in myeloma, lymphoma, and leukemia patients (80). In the development of new therapies, the most progress has been made in the treatment of B-cell leukemia/lymphoma. These account for over 80% of all non-Hodgkin lymphomas (NHL). The current standard treatment is a combination of the anti-CD20 antibody rituximab and chemotherapy, e.g., the CHOP regimen (rituximab, cyclophosphamide, doxorubicin, vincristine, and prednisone) (81, 82). In contrast, NHL/leukemia of T-cell origin remains a therapeutic challenge, and treatment advances lag behind those for B-NHL. For example, treatment outcome is worse in pediatric T-ALL patients than in pediatric B-ALL patients (83). Adult T-ALL poses even greater treatment difficulties and no current option prolongs survival satisfactorily (84). In both B- and T-NHL/leukemia patients, outcomes of relapsed disease are usually dismal. Late effects and systemic toxicities related to conventional strategies (chemo- and radiotherapy) must also be considered. This spells out a continuing need for innovative approaches, especially for patients with relapsed B-NHL or newly diagnosed/relapsed T-NHL. Targeted therapies (85), immunotherapy (86), and oncolytic virotherapy have triggered growing interest and are the focus of much attention. Two OVs, the wild-type reovirus and an engineered measles virus, have successfully reached the clinical testing phase (87).^1^^,^^2^ In particular, a non-randomized phase I study conducted in Switzerland and involving cutaneous T-cell lymphoma (CTCL) patients with accessible lesions allowing intratumoral measles virus application has already yielded promising results as regards both the safety and efficacy of this OV treatment (87). One should note, however, that OV trials currently recruiting hematological cancer patients are restricted to refractory MM and that they are strikingly fewer than, for instance, melanoma or glioma OV trials. Given the promising preclinical data that demonstrate the potential of several other OVs to induce oncolytic effects in myeloid, B- and T-cell lymphoma/leukemia models, further clinical development of this anticancer approach is to be expected, and also hoped for, in the case of hematological malignancies. A recent study by Kishore and Kishor, comparing mortality rates between parvovirus-B19-infected and uninfected pediatric ALL patients has raised the intriguing hypothesis that natural B19 infection may exert unexplored oncolytic effects (88).

## Oncolytic H-1PV as a Candidate for Further Development in Oncohematology

After the first demonstration that H-1PV could induce efficient BL cell killing *in vitro* (77, 78) and BL regression in animal models (79), the question arose: might H-1PV be used against other types of hematological cancers? This question is of general interest, since BL is mostly seen in Uganda and Nigeria and is a rare condition outside Africa (89). It prompted us to conduct further studies to assess the capacity of this virus to target cells derived from other hematological malignancies. A panel of commercially available ATCC cell lines derived from aggressive or indolent lymphomas/leukemias of B- or T-cell origin was tested *in vitro* (A. Angelova, Z. Raykov, J. Rommelaere, unpublished data). First, encouraging results were obtained as shown in Table 2. Only one mixed type B-cell lymphoma and one Sézary syndrome CTCL were resistant to H-1PV-induced cell death. This resistance was associated with either the absence (Hut78 cells) or a low level (Farage cells) of progeny virion production and was not due to blockage of virus entry. In contrast, large B-cell-lymphoma-derived cells supported high levels of H-1PV progeny virion production and were almost totally eradicated by very low virus doses. Notably, DLBCL cell lines (e.g., Pfeiffer) with upregulated expression of aldehyde dehydrogenase 1A1 conferring CHOP resistance (90) were among the most sensitive H-1PV targets. These results suggest a potential use of H-1PV in chemoresistant DLBCL cases. Furthermore, H-1PV was able to replicate in T-ALL and some CTCL cells, with striking cytopathic effects. Although CTCL is a relatively rare condition, its incidence has increased about threefold over the last 2–3 decades in the United States (91) and in other regions of the world (92). Advanced disease stages with blood involvement require systemic therapies and, in general, the quality of life of CTCL patients is greatly affected. We are, therefore, now expanding the panel of *in vitro* models to test the antineoplastic potential of H-1PV in several, mostly T-cell-derived, types of hematological cancers, including CTCL. The failure of CHOP-based chemotherapies in CTCL patients has led to the development and subsequent FDA approval of two histone deacetylase inhibitors (HDACis), vorinostat and romidepsin (93–95). As patients often fail to reach or sustain a 50% partial response to these drugs, other agents have to be added in a combinatorial manner, in order to overcome resistance to HDACi (94). OVs, notably H-1PV, appear as potential candidates, as it was recently shown by Li et al. that another HDACi, valproic acid, when combined with oncolytic H-1PV, increases parvovirus-mediated cytotoxicity toward cervical and pancreatic cancer cells, thus resulting in synergistic killing (96). Further preclinical studies are worth conducting to determine whether these findings can be extended to CTCL and other clinically challenging T-cell malignancies such as T-ALL. Interestingly, it was recently reported that expression of the transcription factor TAL-1 (associated with poor prognosis in T-ALL) is markedly downregulated upon HDAC inhibition (97).

**Table 2:** **Responsiveness of lymphoma- and leukemia-derived cell lines to oncolytic H-1PV infection**.

Cell line		Disease	H-1PV-induced killing/sensitivity^a^	H-1PV progeny virion production^b^
**B-cell malignancies**				

Farage	ATCC^®^ CRL-2630™	B-lymphoblast NHL (mixed type)	Resistant	+
Toledo	ATCC^®^ CRL-2631™	DLBCL	++	++
Pfeiffer	ATCC^®^ CRL-2632™	DLBCL	+++	++
DB	ATCC^®^ CRL-2289™	B-lymphoblast large cell lymphoma	+++	+++
RL	ATCC^®^ CRL-2261™	B-lymphoblast NHL	+	++

**T-cell malignancies**				

CCRF-CEM	ATCC^®^ CCL-119™	T-ALL	++	++
Loucy	ATCC^®^ CRL-2629™	T-ALL	+	+
SUP-T1	ATCC^®^ CRL-1942™	T-lymphoblast NHL	+	++
Hut78	ATCC^®^ TIB-161™	CTCL (Sézary syndrome)	Resistant	No
HH	ATCC^®^ CRL2105™	CTCL	+++	++

**Myeloid malignancies**				

HL-60	ATCC^®^ CCL240™	Acute promyelocytic leukemia	++	+

**Malignancies of undetermined cellular origin**				

SR	ATCC^®^ CRL-2262™	Large cell immunoblastic lymphoma	+++	n.a.

*^a^Sensitivity to H-1PV-induced killing is scored as +++, ++, and + when the virus dose required to cause death of 50% of the cells was <5, 5–10, or 10–50 plaque-forming units (pfu)/cell, respectively. Cells were considered “conditionally resistant” when the virus dose required to achieve 50% cell death exceeded 50 pfu/cell*.

*^b^The capacity for H-1PV progeny virion production was scored as +++, ++, or +, when the ratio of the virus titer 72 h postinfection to the titer 12 h postinfection was >100, 10–100, or <10, respectively*.

*NHL, non-Hodgkin lymphoma; DLBCL, diffuse large B-cell lymphoma; T-ALL, T-cell acute lymphoblastic leukemia; CTCL, cutaneous T-cell lymphoma; n.a., not analyzed*.

## Conclusion and Perspective

In conclusion, oncolytic H-1PV has shown outstanding oncosuppressive activity in preclinical models of various solid tumors. Data from the first H-1PV clinical trial in recurrent glioblastoma patients have confirmed the excellent safety and tolerability of this virus upon local or systemic application. Accumulating preclinical evidence shows, furthermore, that H-1PV can efficiently kill, *via* productive infection, cancer cells derived from different hematological malignancies. These include both rituximab- and chemotherapy-resistant B-cell lymphomas and T-cell leukemia/lymphoma, which currently pose a major therapeutic challenge. These first results strongly encourage further preclinical studies aimed at substantiating the oncolytic and adjuvant potential of H-1PV against hematological cancers, both as single agent and as a component of combination treatments. These studies should pave the way toward innovative improvements of current standard therapies, for the benefit of chemotherapy-resistant and relapsing patients.

## Author Contributions

AA, MW-H, JR, and AG contributed to writing the present article. AA contributed to generating, analyzing, and presenting the unpublished data.

## Conflict of Interest Statement

The authors declare that the research was conducted in the absence of any commercial or financial relationships that could be construed as a potential conflict of interest.

## Footnotes

^1^*Oncolytics Biotech Inc. – Clinical Trials*. Available from: http://www.oncolyticsbiotech.com/reolysin/clinical-trials/

^2^*ClinicalTrials.gov: Vaccine Therapy With or Without Cyclophosphamide in Treating Patients With Recurrent or Refractory Multiple Myeloma*. Available from: https://clinicaltrials.gov/ct2/show/NCT00450814

## Abbreviations

ALL, acute lymphoblastic leukemia; BL, Burkitt’s lymphoma; OV(s), oncolytic virus(es); H-1PV, parvovirus H-1; PDAC, pancreatic ductal adenocarcinoma; MM, multiple myeloma; CV, coxsackievirus; VSV, vesicular stomatitis virus; NHL, non-Hodgkin lymphoma; R-CHOP, rituximab, cyclophosphamide, doxorubicin, vincristine, prednisone; DLBCL, diffuse large B-cell lymphoma; CTCL, cutaneous T-cell lymphoma; HDACi, histone deacetylase inhibitor.
